# Impact of Superficial Keratectomy on Corneal Topography, Aberration, and Densitometry in Salzmann Nodular Degeneration

**DOI:** 10.3390/jcm15010178

**Published:** 2025-12-26

**Authors:** Ziqiao Qi, Ritika Mukhija, Gabriella Quiney, Mayank A. Nanavaty

**Affiliations:** 1 Sussex Eye Hospital, University Hospitals Sussex NHS Foundation Trust, Eastern Road, Brighton BN2 5BF, UK; 2 Brighton & Sussex Medical School, University of Sussex, Falmer, Brighton BN1 9PX, UK

**Keywords:** Salzmann nodular degeneration, corneal aberrations, corneal topography, corneal densitometry

## Abstract

**Objectives**: The aim of this study was to evaluate changes in corneal topography, wavefront aberrations, and densitometry after superficial keratectomy (SK) for Salzmann nodular degeneration (SND). **Methods**: This was an observational retrospective study. Pre- and post-operative data, including best spectacle-corrected visual acuity (BCVA), refraction, and Pentacam^®^ topography scans, were analysed. Changes in keratometry (K), wavefront aberrations, and corneal optical densitometry (COD) were evaluated. **Results**: A total of 21 eyes of 17 patients who underwent SK for SND were included. At an average follow-up of 6.3 ± 4.4 months, there was a significant reduction in RMS (root mean square) values for total wavefront aberrations after surgery (mean reduction: −3.89 ± 4.08 μm, *p* = 0.001), lower-order aberrations (mean reduction: −3.47 ± 3.52 μm, *p* = 0.001), and higher-order aberrations (mean: −0.42 ± 0.58 μm, *p* < 0.001). There was a significant improvement in BCVA (mean change: 0.27 ± 0.31 logMAR, *p* < 0.001) and spherical equivalent (mean change: −2.09 ± 2.73 D, *p* = 0.002), and a reduction in refractive cylinder (mean reduction: −0.85 ± 1.14 D, *p* = 0.008). There was a significant reduction in corneal astigmatism (mean reduction 1.04 ± 2.88 D; *p* = 0.041), and an increase in flat keratometry (mean change 1.46 ± 3.10 D; *p* = 0.029). Lastly, there was a significant decrease in total COD values in all zones except for the central 2 mm (*p* < 0.05) and in the overall total 12 mm cornea (*p* = 0.035). **Conclusions**: SK for SND resulted in better visual acuity and potentially improved quality as suggested by the reduction in corneal aberrations and improvement in corneal regularity and transparency.

## 1. Introduction

Salzmann nodular degeneration (SND) is a degenerative, non-inflammatory, slowly progressive corneal pathology characterised by hypertrophic nodules in the corneal epithelium [1]. These nodules typically range from 1 to 3 mm in size but can vary in presentation, affecting one or both eyes and appearing as single lesions or as multiple formations [2]. SND typically starts as an innocuous, asymptomatic condition. Still, its progression may cause visual disturbances due to astigmatism and central corneal flattening-induced hyperopia, as well as epithelial irregularities resulting in recurrent erosions, photophobia, and tearing [3]. While the exact aetiology of SND remains uncertain, it is commonly associated with chronic ocular irritation and inflammation. For instance, ophthalmic pathologies such as trachoma, interstitial keratitis, vernal keratoconjunctivitis, ocular trauma, and prior ocular surgeries are frequently implicated as contributing factors in the formation of SND nodules, as they cause persistent irritation of the corneal epithelium [1].

Surgical intervention may be required for patients presenting with symptomatic SND. In most cases, minimally invasive procedures such as a Salzmann nodule peel or nodulectomy for localised nodules, or a superficial keratectomy (SK) for multiple nodules or a larger area of involvement, are appropriate. More severe cases, especially those with ocular comorbidities resulting in corneal scarring, such as trachoma, may require keratoplasty, either penetrating or anterior lamellar [4]. Restoring a smoother corneal surface, which is conducive to natural re-epithelialisation, helps reduce pain and photophobia and improve visual outcomes [5]. The current literature has limited reports evaluating the impact of SK on corneal topography and aberrations. This study aims to describe and quantify any changes in wavefront aberrations, corneal topography, and corneal optical densitometry following SK for Salzmann nodular degeneration.

## 2. Materials and Methods

This is an observational study conducted at a single tertiary care eye hospital. Data was retrospectively retrieved from the hospital’s operating theatre records and corresponding patient notes for those who underwent surgery between 2019 and 2024. Inclusion criteria were patients who had undergone Salzmann nodular peel or superficial keratectomy (SK) for Salzmann nodular degeneration (SND) and had both pre-operative and post-operative corneal topography scans available. Scans were typically performed in clinics around six weeks to three months post-surgery. Corneal topography data were obtained using the Pentacam^®^ system (OCULUS, Inc., St. Louis, MO, USA), a high-resolution, Scheimpflug-based device for assessing corneal curvature, aberrations, and densitometry.

In this study, both Salzmann nodular peel and superficial keratectomy are referred to as the latter (SK); the former involves manual peeling of the nodule with surrounding epithelium, and the latter involves alcohol-assisted manual epithelial debridement using a sterile sponge or surgical blade. Following epithelial debridement, the cornea was irrigated with balanced salt solution, and a bandage contact lens was often placed for comfort. Patients were discharged with G. ofloxacin four times a day for 2 weeks and G. prednisolone four times a day for two weeks, along with topical G. sodium hyaluronate 0.2% on an as-needed basis for 2 weeks. Patients were followed up at 2 weeks and between 6 and 12 weeks. Post-operative corneal topography scans were typically performed at the subsequent follow-up, typically ranging from 6 weeks to 6 months.

The primary outcome measure was the change in wavefront aberrations (WFAs) as measured by analysis of total aberrations, lower-order aberrations (LOAs), and higher-order corneal aberrations (HOA) expressed in root mean square (RMS) after surgery. The secondary outcome measures were changes in best-corrected visual acuity (BCVA), refractive cylinder, spherical equivalent, keratometry, and corneal densitometry.

**Figure 1:**
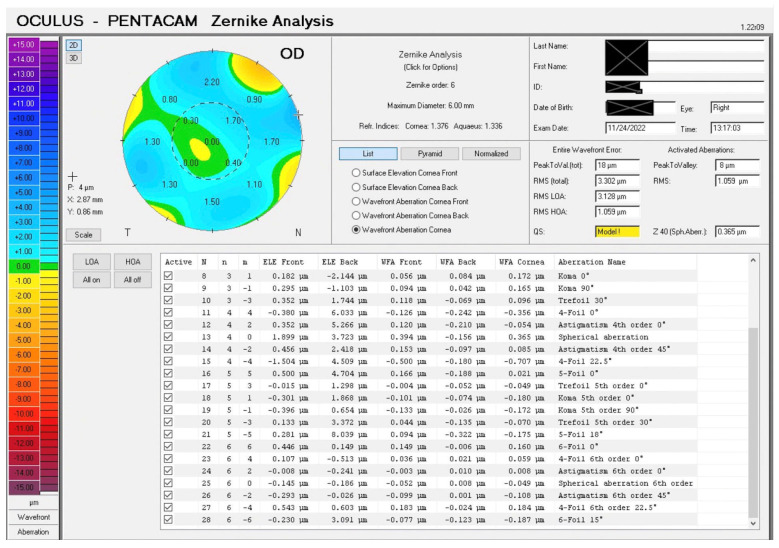
Sample Zernike wavefront analysis on Pentacam Scheimpflug imaging.

In addition to the lower, higher-order and total aberrations, individual aberrations from the Zernike wavefront analysis were also evaluated (Figure 1). Keratometry (K) readings were obtained using the 4-map refractive analysis and Holladay Equivalent Keratometry Reading (EKR) report. The following parameters were recorded: central flat K (K1), central steep K (K2), and central average K (K mean) using the former, and flat, steep, and mean K values, respectively, in different zones (1 mm, 2 mm, 3 mm, 4 mm, 4.5 mm, 5 mm, 6 mm, 7 mm) from the latter report. Corneal optical densitometry values were recorded to assess changes in corneal transparency, with measurements stratified by zone (0–2 mm, 2–6 mm, 6–10 mm, 10–12 mm) and depth (e.g., anterior, central, posterior).

Pentacam utilises the rotating Scheimpflug principle to provide data on the anterior and posterior corneal topography and elevation, pachymetry, anterior chamber parameters, pupil size, and other indices. All scans were taken in the same room in standardised mesopic light conditions for all participants. For Pentacam HR^®^ (Wetzlar, Germany) measurements, subjects were asked to look at the fixation target. The machine automatically commenced its measurements when it detected correct alignment with the corneal apex and achieved optimal focus. The Pentacam HR measurement protocol includes a series of 25 images (1003 × 520 pixels) taken over different meridians using a constant blue light source. The Pentacam HR^®^ Scheimpflug tomography system uses a high-resolution, 1.45-megapixel camera. The camera captures 138,000 data points in less than 2 s. A 475 nm wavelength blue light-emitting diode and a Scheimpflug camera rotate together around the optical axis to acquire images of the anterior segment of the eye [6,7]. The Scheimpflug system provides the K values and orientation meridian of the steepest keratometry (MoSK). The detailed Holladay Equivalent K report of this machine gives equivalent keratometry at various optical scan diameters. For this study, the keratometric values for 4.5 mm, 5 mm, 6 mm, and 7 mm optical zones were entered from the detailed Holladay Equivalent K reading report [7]. Pentacam HR^®^ automatically converts the corneal elevation profile into corneal wavefront data using the Zernike polynomials with an expansion up to the 10th order. The Zernike terms are defined at a maximum diameter of 6 mm [8]. We used Pentacam software version 1.20r36 in this study.

In addition to the corneal topographic assessments, Scheimpflug images on the Pentacam HR^®^ provide information on corneal optical densitometry (COD). Densitometry values were obtained and expressed in standardised greyscale units (GSUs). The GSU scale is calibrated by averages of proprietary software, which defines a minimum light scatter of 0 (maximum transparency) and a maximum light scatter of 100 (minimum transparency). To proceed with local densitometry analysis, measurements were taken within the default 12 mm diameter area, which is subdivided into four concentric radial zones. The corneal apex was indicated as the central point of all zones:

Zone 1: The first central zone is 2 mm in width.

Zone 2: The second annulus extends from 2 to 6 mm.

Zone 3: The third zone annulus goes from 6 to 10 mm in diameter.

Zone 4: The final external annulus is from 10 to 12 mm in diameter.

These topographical zones are established in the software. Furthermore, in the software, the COD was analysed in different depths:

a. Anterior layer with 120 µm of the outermost cornea.
b. The mid-stromal layer is between the anterior and posterior layers.
c. Posterior layer with a thickness of 60 µm.

Statistical analysis was performed using Microsoft Excel Version 16.89.1. Mean, standard deviation, minimum, and maximum values were calculated for all parameters. To evaluate the pre- and post-operative differences in the various parameters, statistical analysis was performed using linear mixed models with random intercept for patient ID, utilising Restricted Maximum Likelihood (REML) estimation. This approach accounts for repeated measurements within subjects and provides more robust inference than standard paired *t*-tests. It was used as our dataset contains two eyes from the same patient in some cases. A *p* value of less than 0.05 was considered statistically significant, and all values are reported up to three decimal places.

## 3. Results

A total of 21 eyes from 17 patients diagnosed with Salzmann nodular degeneration (SND) were included in this observational study. The mean age was 64.8 ± 13.3 years (range: 47–93), with 7 male and 10 female patients. Common ocular comorbidities were cataract (n = 6), meibomian gland dysfunction (n = 2), rosacea, and previous trauma (n = 2). Table 1 presents the characteristics of the patients in this study. The most common presentation was ocular surface symptoms like soreness, discomfort, or foreign body sensation (n = 10), followed closely by blurred vision (n = 9), whereas a small proportion were either asymptomatic (n = 1) or had resulting recurrent corneal erosions (n = 1). The most common location of nodules was paracentral (n = 8), followed by mid-peripheral (n = 7); some eyes had a combination of both (n = 4) and a minority had mid-peripheral and peripheral nodules (n = 2). None of the cases included in the study were recurrent SND and, therefore, as a departmental protocol, mitomycin C was not used in any of the surgeries. Average follow-up duration at which the Pentacam topography scan was captured was 6.3 ± 4.4 months (range 2–15 months).

### 3.1. Primary Outcome Measure

There was a significant reduction in lower-order, higher-order, and total wavefront aberrations (WFAs) after surgery, as shown in Table 2. Total RMS aberrations showed significant improvement post-operatively (mean reduction: −3.89 ± 4.08 μm, *p* = 0.001). Lower-order aberrations (LOAs) demonstrated a significant decrease (mean reduction: −3.47 ± 3.52 μm, *p* = 0.001), indicating improved defocus and astigmatism correction. Higher-order aberrations (HOAs) showed a modest but significant reduction (mean: −0.42 ± 0.58 μm, *p* < 0.001), suggesting improved optical quality with fewer coma and spherical aberration components. Specific aberrations that showed a statistically significant reduction after surgery were front and total aberrations in **Z_1_^1^** (tilt in X or horizontal tilt) and **Z_3_^1^** (horizontal coma) and the total aberrations in **Z_2_^−2^** (primary oblique astigmatism) (Table 2). There was also a significant change in back aberrations in **Z_4_^2^** (fourth-order astigmatism, 0°), and in the front aberrations in **Z_3_^−1^** (vertical coma), though the latter was just short of reaching statistical significance (*p* = 0.05). Detailed wavefront analysis is presented in Supplementary Table S1.

### 3.2. Secondary Outcomes

(a) *Visual Acuity, refractive cylinder, and spherical equivalent:*

Individual pre- and post-operative BCVA values are described in Table 1. Fourteen patients had an improvement in their BCVA after surgery. The mean pre-operative best spectacle-corrected visual acuity (BCVA) was logMAR 0.27 ± 0.27 (range: 1.2 to 0), the mean pre-op refractive cylinder was −2.09 ± 1.55 D (range: −5.50 to 0.25), and the mean spherical equivalent was 0.96 ± 2.73 D (−5.13 to 4.50). These changed post-operatively to a mean BCVA of LogMAR 0.14 ± 0.14 (range: 0.40 to 0), mean refractive cylinder of −0.90 ± 0.91 D (range: −2.50 to +0.50), and mean spherical equivalent of −0.43 ± 2.40 D (range: −7.00 to +1.75). BCVA improved significantly from pre-operative to post-operative assessment (mean improvement: 0.27 ± 0.31 logMAR, *p* < 0.001), representing approximately 2–3 lines of visual acuity on the Snellen chart. Similarly, spherical equivalent showed significant refractive correction (mean change: −2.09 ± 2.73 D, *p* = 0.002), with high myopes demonstrating greater refractive shifts, and refractive cylinder decreased significantly (mean reduction: −0.85 ± 1.14 D, *p* = 0.008), indicating improved astigmatism management. Our results demonstrate a statistically significant improvement in visual acuity, spherical equivalent, and refractive astigmatism.

(b) *Keratometry:*

On the four-map refractive analysis, there was a significant increase in flat keratometry or K1 from 40.14 ± 2.69 D to 41.55 ± 2.68 D (mean change 1.46 ± 3.10 D; *p* = 0.029), and a significant reduction in corneal astigmatism from 3.31 ± 2.05 D pre-operatively to 1.56 ± 1.73 D post-operatively (mean reduction 1.04 ± 2.88 D; *p* = 0.041). Mean keratometry values changed from 41.88 ± 2.07 D to 42.65 ± 2.15 D, though the results were short of reaching statistical significance (mean change 0.60 ± 2.15 D; *p* = 0.077), whilst the steep keratometry or K2 (43.62 ± 1.84 D to 43.74 ± 1.89 D, mean change 0.37 ± 1.89 D; *p* = 0.224) did not show any significant change. A similar trend of increase in K1 values was also seen on the Holladay EKR report in zones 4.5 mm, 5 mm, 6 mm, and 7 mm (Table 3). Further, there was a significant flattening of K2 or steep keratometry in zones 3 mm and 7 mm, while there was no difference in the other K values in zones 3 mm to 7 mm or in any of the K values within the central 2 mm zones; detailed analysis is presented in Supplementary Table S2.

(c) *Corneal Optical Densitometry (COD):*

There was a significant decrease in total COD values in zones 2–6 mm, 6–10 mm, 10–12 mm, and in the overall total 12 mm, as shown in Table 3, indicating improved transparency and reduced scattering. While there was a decrease in COD values across all zones and at all depths, the difference was not statistically significant; detailed analysis is presented in Supplementary Table S3.

Finally, in order to evaluate whether optical quality or corneal transparency metrics predicted visual outcome, mixed effects linear regression models were constructed with logMAR BCVA as the dependent variable, including patient as a random effect and pre-operative BCVA, time (pre/post), and either total RMS or central densitometry (0–2 mm, total) as fixed effects. In the RMS model, total RMS was not associated with BCVA (β ≈ 0.00 logMAR per μm, *p* = 0.997), whereas the time effect remained highly significant (β ≈ −0.97, *p* = 0.001). Similarly, in the densitometry model, 0–2 mm densitometry was not a significant predictor (β ≈ −0.005 logMAR per GSU, *p* = 0.790), with a persistent time effect (β ≈ −0.90, *p* = 0.016). A combined model including both total RMS and densitometry did not change these findings; neither parameter independently predicted BCVA (*p* > 0.750 for both).

## 4. Discussion

Our study of 21 eyes that underwent superficial keratectomy (SK) in Salzmann nodular degeneration (SND) yielded coherent, modality-spanning improvements across visual function, anterior corneal optics, keratometric regularity, and localised tissue clarity. Taken together, the findings support an anatomy-to-optics-to-function pathway: removing nodular elevations primarily regularises the anterior corneal surface, reduces directionally biased lower- and higher-order aberrations (notably horizontal tilt, horizontal coma, and oblique astigmatism), and improves best-corrected visual acuity (BCVA), with corneal densitometry changes playing a supportive and region-specific role. This mechanistic framing is consistent with the known pathology of SND and aligns with the published literature on SK and surface ablation approaches for anterior corneal irregularity [3,4,5,9,10]. The linear mixed model analysis demonstrates significant improvements across both primary and secondary outcome measures following corneal surgery.

There were significant reductions in corneal wavefront aberrations across the whole spectrum following SK, indicating improved optical quality of the cornea. These findings align with and expand upon the results of a case reported by Roszkowska et al., where they reported a significant decrease in total aberrations and HOAs using root mean square (RMS) values following manual alcohol-assisted removal of SND [9]. However, we noted a significant reduction across the spectrum and in both lower- and higher-order aberrations and our study provides a more in-depth analysis of individual wavefront errors up to the fourth order as reported in the Zernike analysis. The greater reduction in LOAs compared to HOAs suggests effective correction of defocus and astigmatism while maintaining corneal optical quality. There was a significant change in horizontal tilt (Z_1_^1^), primary oblique astigmatism (Z_2_^−2^), and horizontal coma (Z_3_^1^) in our study. Salzmann nodules are characteristically elevated, subepithelial lesions that produce localised asymmetry on the anterior surface. The resulting optical path distortion is captured in coma-like and tilt-like aberrations, which degrade retinal image quality out of proportion to spherical or cylindrical refractive error. By physically removing the nodule(s), SK restores a more symmetric anterior dome, lowers these WFAs, and permits improved BCVA and subjective vision quality. Reports assessing higher-order aberrations before and after anterior surface smoothing—whether in SND, epithelial basement membrane dystrophy, or post-keratoplasty irregularities—frequently note reductions in coma-like terms as the predominant optical improvement [10,11,12,13]. The observed decrease in primary oblique astigmatism in our study correlates with improved keratometric regularity and better axis-consistent optics, with a tighter distribution of corneal power and reduced directional irregularity that may be hard to neutralise with standard refraction.

Across the cohort, SK was associated with a BCVA improvement of 0.27 logMAR, representing clinically meaningful visual gain (Table 1). Further, there was a significant reduction in both spherical equivalent and refractive cylinder, indicating improvement in overall refractive correction following the procedure, though it is important to note that spherical equivalent may be impacted by other co-morbidities like cataract. This is in line with other studies of SK and phototherapeutic keratectomy (PTK) in SND that report improved BCVA and reduced irregular astigmatism [5,10,11,12]. In a recent literature review on superficial keratectomy, Salari et al. [14] found that out of six case series treating SND with SK, only one series reported enhancement of BCVA following SK. All other studies reported that BCVA did not significantly change post-operatively. However, all the studies that reported changes in refractive astigmatism reported reduced astigmatism post-operatively. On the other hand, Raber and Eagle [15], who reported 30 eyes that underwent SK, saw an enhanced best spectacle-corrected visual acuity and reduced visual symptoms post-operatively. These mixed results can be explained in part by the small number of SND cases, both in our study and in the literature, and because in some cases SND may only reduce vision by inducing astigmatism, which is fully correctable by glasses.

Our study demonstrated a statistically significant steepening in keratometry values after treatment, highlighted both by a significant increase in the central flat keratometry (K1) from 40.14 D to 41.55 D (*p* = 0.029), and also across zones 4.5 mm, 5 mm, 6 mm, and 7 mm. These findings corroborate well with the common location of Salzman nodules in our study, which was either paracentral or mid-peripheral, and as the nodules cause flattening of the corneal curvature, their removal manifests with steepening in these areas. This is supported by Goerlitz-Jessen et al., who also reported a significant increase in mean K values (*p* = 0.02) as part of their research on the effect of SND on biometry measurements [16]. Furthermore, there was a significant flattening in steep K values in zones 3 mm and 7 mm and an overall increase in corneal regularity as highlighted by a significant reduction in keratometric astigmatism (*p* = 0.003). Not only does this underscore the significant impact SND can have on keratometry measurements, but it also highlights the importance of treating SND prior to cataract surgery to obtain accurate biometric data. This is because the cornea accounts for approximately two-thirds of the eye’s total refractive power, making keratometry measurements crucial for accurate intraocular lens (IOL) power calculations in patients with coexisting cataracts [17]. Keratometric regularisation after SK is well documented, particularly in cases with paracentral nodules, where topographic asymmetry and higher-order aberrations co-localise [18,19,20].

Corneal optical densitometry (COD) measures the light-scattering properties of the cornea, with higher values indicating reduced transparency [21]. In SND, the presence of nodules increases light scatter by introducing additional surface irregularities, thereby diminishing transparency and visual clarity [22]. Our COD analysis reported evident changes across the entire cornea and across all depths, but these were statistically significant only when analysed across the total corneal depth for all zones except the central 2 mm. These results indicate changes mapping to the nodule region as paracentral, mid-peripheral and peripheral, rather than indicating a diffuse, global decrease across all annuli; however, these were significant enough to warrant a reduction in the overall values when averaged across the entire 12 mm of cornea. The anatomical and optical logic observed here aligns with the established pathophysiology and management of SND. These findings align with those reported by Otri et al. in their study of corneal densitometry as an indicator of corneal health in bacterial keratitis [23]. COD, as a global metric, is often less sensitive to focal lesion removal; changes tend to be greatest in the annulus and the layer overlapping the pathology [24,25,26,27]. This matches our localised COD improvements and reinforces that WFA and keratometry are the most direct surrogates for functional gain in focal anterior disorders.

Though our study demonstrated improvement across visual acuity, wavefront aberrations, and corneal transparency, mixed effects linear regression models revealed that both aberrations (total RMS) and corneal optical densitometry did not independently predict BCVA (*p* > 0.75 for both). These exploratory mixed effects analyses suggest that, within the limitations of the small sample size and incomplete post-operative data, the magnitude of residual wavefront error and corneal densitometry does not independently determine best-corrected acuity. The strong fixed effect of time indicates that the major driver of BCVA improvement is the surgery itself (removal of the lesion and associated refractive change), whereas fine variations in post-operative optical quality and transparency, as captured by global RMS and 0–2 mm densitometry, contribute little additional explanatory power. Larger datasets with more complete paired measurements will be required to determine whether localised or higher-order aberration components, or layer-specific densitometry, show a closer relationship with functional visual outcomes.

The results of our study have several clinical implications for clinicians managing SND. The LMM approach appropriately accounted for the hierarchical data structure, providing robust estimates that incorporate within-patient and within-eye correlations. The significant random effects variance components confirm the importance of accounting for patient-level clustering in the analysis. Earlier consideration of SK is reasonable in patients with visually significant irregular astigmatism, symptomatic blur, fluctuating vision quality, or contact lens intolerance, when surface irregularity is suspected as the driver. A key strength of this study is its multimodal assessment, which includes BCVA, keratometry, wavefront aberrations stratified by front versus total cornea, and densitometry, allowing for a unified, causal narrative that spans anatomy, optics, and function. Limitations include the retrospective design of the study, the resulting variable follow-up duration, and the cohort size. Measurement sensitivity to tear film quality and fixation can introduce noise into both WFA and densitometry data; however, paired designs mitigate some variability, and residual noise could attenuate observed effect sizes. Follow-up length also matters; epithelial remodelling and subtle stromal changes evolve over weeks to months, and the long-term stability of WFA and densitometry reductions—not just BCVA—warrants longitudinal assessment. Future studies may shed more insight into this.

## 5. Conclusions

In conclusion, our study reinforces superficial keratectomy as an effective surgical intervention for visually significant Salzman nodular degeneration, resulting in better visual acuity and potentially improved quality of vision as can be suggested by a reduction in corneal aberrations and an improvement in corneal regularity and transparency.

## Figures and Tables

**Table 1:** Patient characteristics.

Age/Sex	Presenting Symptom or Indication for Surgery	Location of Nodule(s)	Pre-op LogMAR BCVA	Post-op LogMAR BCVA
75/M	Blurred vision	Paracentral	0.48	0.30
75/M	Blurred vision	Paracentral	1.20	0.40
58/F	Foreign body sensation	Mid-peripheral	0.00	0.00
65/F	Blurred vision	Paracentral	0.30	0.00
66/F	Blurred vision	Mid-peripheral	0.10	0.00
48/M	Foreign body sensation	Mid-peripheral	0.18	0.10
63/M	Blurred vision	Mid-peripheral	0.18	0.18
63/M	Blurred vision	Paracentral	0.18	0.18
67/M	Soreness, epiphora	Paracentral	0.18	0.10
48/F	Foreign body sensation	Paracentral and mid-peripheral	0.18	0.00
77/M	Foreign body sensation	Paracentral and mid-peripheral	0.30	0.15
69/F	Blurred vision, astigmatism	Paracentral	0.48	0.30
52/M	Blurred vision	Paracentral and mid-peripheral	0.48	0.00
47/F	Foreign body sensation	Mid-peripheral	0.00	0.00
47/F	Foreign body sensation	Mid-peripheral	0.15	0.10
66/M	Asymptomatic	Paracentral	0.15	0.15
93/F	Foreign body sensation	Mid-peripheral and peripheral	0.50	0.30
93/F	Foreign body sensation	Mid-peripheral and peripheral	0.50	0.40
67/F	Blurred vision, astigmatism	Paracentral and mid-peripheral	0.15	0.00
53/F	Recurrent corneal erosions	Paracentral	0.00	0.00
68/F	Soreness, discomfort	Mid-peripheral	0.00	0.00

F: Female; M: Male.

**Table 2:** Pre- and post-op Zernike’s wavefront analysis depicting changes in overall aberrations and specific aberrations with a statistically significant difference.

Parameter	Location (WFA)	Pre-op (μm) Mean (±SD)	Post-op (μm) Mean (±SD)	*p*-Value
RMS Total	-	7.439 (±3.648)	4.283 (±2.076)	0.001
RMS Lower-Order Aberrations (LOAs)	-	6.858 (±3.524)	3.965 (±1.830)	0.001
RMS Higher-Order Aberrations (HOAs)	-	2.792 (±1.194)	1.515 (±1.146)	0.000
**Z_1_^1^** Horizontal Tilt (Tilt in X)	Front	−1.622 (±3.978)	0.409 (±1.627)	0.038
	Total	−1.596 (±4.431)	0.451 (±1.734)	0.047
**Z_2_^−2^** Primary Oblique Astigmatism	Total	−0.634 (±2.853)	0.190 (±1.664)	0.032
**Z_3_^1^** Horizontal Coma	Front	−0.381 (±1.173)	0.191 (±0.575)	0.048
	Total	−0.373 (±1.296)	0.245 (±0.663)	0.048

**Table 3:** Keratometry and corneal optical densitometry analysis demonstrating values with statistically significant difference between pre- and post-op.

Keratometry from the Holladay EKR Report			
Zone (mm)/Keratometry Meridian	Pre-op Mean (±SD) (D)	Post-op Mean (±SD) (D)	*p*-Value
3 mm/K2 (Steep K)	43.08 (±2.72)	42.79 (±2.32)	0.034
4.5 mm/K1 (Flat K)	40.17 (±2.52)	41.07 (±3.03)	0.018
5 mm/K1 (Flat K)	40.23 (±2.48)	41.20 (±3.05)	0.003
6 mm/K1 (Flat K)	40.56 (± 2.30)	41.59 (±3.06)	<0.001
7 mm/K1 (Flat K)	40.89 (±2.23)	41.83 (±3.13)	<0.001
7 mm/K2 (Steep K)	44.60 (±2.59)	43.97 (±1.91)	0.037
**Corneal Optical Densitometry (COD)**			
**Zone/Depth**	**Pre-op Mean (±SD) (µm)**	**Post-op Mean (±SD) (µm)**	***p*-Value**
2–6 mm/Total	18.76 (±6.46)	15.73 (±4.27)	<0.001
6–10 mm/Total	30.79 (±10.90)	26.25 (±8.17)	0.026
10–12 mm/Total	33.47 (±9.66)	28.46 (±5.83)	0.002
Overall cornea/Total depth or all zones	24.85 (±7.23)	20.94 (±4.90)	<0.001

## Data Availability

Data is contained within the article or Supplementary Material. The original contributions presented in this study are included in the article/Supplementary Material. Further inquiries can be directed to the corresponding author.

## Supplementary Materials

The following supporting information can be downloaded at https://www.mdpi.com/article/10.3390/jcm15010178/s1: Table S1: Detailed pre- and post-op Zernike’s wavefront aberration analysis depicting changes up to fourth-order Zernike’s polynomials; Table S2: Detailed pre- and post-op keratometry data from the Holladay EKR report; Table S3: Detailed pre- and post-op corneal optical densitometry analysis.
