# Supplementary material for: Impact of Superficial Keratectomy on Corneal Topography, Aberration, and Densitometry in Salzmann Nodular Degeneration
Source: J Clin Med. 2025 Dec 26;15(1):178. doi: 10.3390/jcm15010178 (PMC12786954; doi:10.3390/jcm15010178)
Supplement: Supplementary file 1 [file jcm-15-00178-s001.zip › jcm-4022228-supplementary.pdf]

Supplementary Table S1: Detailed pre- and post-op Zernike's wavefront aberration analysis depicting changes up to fourth-order Zernike's polynomials

Parameter	Location	Pre-op (μm) Mean (±SD)	Post-op (μm) Mean (±SD)	P-Value
Z_0^0 Piston	WFA front	1.463 (±3.019)	2.579 (±1.400)	0.165
	WFA back	0.372 (±0.490)	-0.694 (±0.370)	0.058
	WFA total	599.176 (±73.406)	574.769 (±47.129)	0.139
Z_1^1 Tilt in X	WFA front	-1.622 (±3.978)	0.409 (±1.627)	**0.038**
	WFA back	0.218 (±0.529)	-0.012 (±0.343)	0.242
	WFA total	-1.596 (±4.431)	0.451 (±1.734)	**0.047**
Z_1^{-1} Tilt in Y	WFA front	-1.219 (±2.48)	0.174 (±1.233)	0.110
	WFA back	0.134 (±0.846)	-0.064(±0.27)	0.346
	WFA total	-1.247 (±2.988)	0.185 (±1.479)	0.064
Z_2^2 Astigmatism 0°	WFA front	-0.102 (±2.494)	-0.599 (±1.897)	0.339
	WFA back	0.160 (±0.568)	0.25 (±0.364)	0.535
	WFA total	0.041 (±2.942)	-0.437 (±2.037)	0.425
Z_2^0 Defocus	WFA front	1.3387 (±2.322)	2.186 (±1.221)	0.177
	WFA back	-0.452 (±0.352)	-0.676 (±0.334)	0.077
	WFA total	1.41 (±2.642)	2.094 (±1.373)	0.320
Z_2^{-2} Oblique Astigmatism 45°	WFA front	2.409 (±2.409)	0.167 (±1.334)	0.247
	WFA back	-0.07 (±0.406)	0.012 (±0.298)	0.399
	WFA total	-0.634 (±2.853)	0.190 (±1.664)	**0.032**
Z_3^3 Trefoil 0°	WFA front	0.250 (±1.237)	0.240 (±0.954)	0.933
	WFA back	0.051 (±0.258)	-0.07 (±0.129)	0.110
	WFA total	0.325 (±1.398)	0.245(±1.034)	0.956
Z_3^1 Horizontal Coma 0°	WFA front	-0.381 (±1.173)	0.191 (±0.575)	**0.048**
	WFA back	0.052 (±0.120)	0.034 (±0.108)	0.823
	WFA total	-0.373 (±1.296)	0.245 (±0.663)	**0.048**
Z_3^{-1} Vertical Coma 90°	WFA front	-0.406 (±0.900)	0.0887 (±0.448)	**0.050**
	WFA back	0.002 (±0.226)	-0.088 (±0.223)	0.101
	WFA total	-0.437 (±1.088)	0.0385 (±0.604)	0.111
Z_3^{-3} Trefoil 30°	WFA front	-0.188 (±0.789)	0.136 (±0.410)	0.219
	WFA back	-0.0219 (±0.338)	-0.017 (±0.173)	0.860
	WFA total	-0.226 (±0.906)	0.141 (±0.450)	0.179
Z_4^4 Horizontal Quadrafoil 0°	WFA front	0.043 (±0.574)	0.08 (±0.482)	0.860
	WFA back	-0.037 (±0.241)	-0.101 (±0.157)	0.351
	WFA total	0.011 (±0.656)	-0.007 (±0.446)	0.918
Z_4^2 Horizontal Astigmatism (4th Order 0°)	WFA front	-0.085 (±0.313)	-0.028 (±0.208)	0.550
	WFA back	-0.054 (±0.089)	-0.004 (± 0.083)	**0.033**
	WFA total	-0.149 (±0.344)	-0.039 (± 0.212)	0.304
Z_4^0 Spherical Aberration	WFA front	0.332 (±0.403)	0.465 (±0.372)	0.256
	WFA back	-0.125 (±0.060)	-0.160 (±0.106)	0.155
	WFA total	0.325 (±0.466)	0.443 (±0.443)	0.382
Z_4^{-2} Oblique Astigmatism (4th Order 45°)	WFA front	0.018 (±0.498)	0.014 (±0.23)	0.917
	WFA back	0.027 (±0.116)	0.027 (±0.082)	0.941
	WFA total	0.048 (±0.534)	0.039 (±0.25)	0.860
Z_4^{-4} Oblique Quadrafoil 22.5°	WFA front	0.007(±0.726)	0.039(±0.361)	0.905
	WFA back	0.002 (±0.198)	-0.020 (±0.091)	0.609
	WFA total	0.008 (±0.879)	0.074 (±0.344)	0.848

Supplementary Table S2: Detailed pre- and post-op keratometry data from the Holladay EKR report

Zone (mm)	Meridian	Pre-op Mean (±SD) (D)	Post-op Mean (±SD) (D)	P-value
1	K1 (Flat K)	39.76 (±4.09)	40.61 (±3.28)	0.369
	K2 (Steep K)	41.33 (±3.91)	41.8 (±3.31)	0.523
	Mean K	40.55 (±3.96)	41.20 (±3.17)	0.441
2	K1 (Flat K)	39.87 (±3.55)	40.59 (±3.37)	0.348
	K2 (Steep K)	42.35 (±2.93)	42.31 (±2.99)	0.793
	Mean K	41.11 (±3.13)	41.46 (±3.06)	0.631
3	K1 (Flat K)	40.07 (±2.99)	40.79 (±3.06)	0.226
	K2 (Steep K)	43.08 (±2.72)	42.79 (±2.32)	**0.034**
	Mean K	41.58 (±2.71)	41.78 (±2.53)	0.862
4	K1 (Flat K)	40.11 (±2.68)	40.95 (±3.05)	0.060
	K2 (Steep K)	43.60 (±2.62)	43.18 (±2.17)	0.051
	Mean K	41.82 (±2.47)	42.07 (±2.46)	0.570
4.5	K1 (Flat K)	40.17 (±2.52)	41.07 (±3.03)	**0.018**
	K2 (Steep K)	43.81 (±2.61)	43.35 (±2.12)	0.080
	Mean K	41.99 (±2.41)	42.17 (±2.43)	0.500
5	K1 (Flat K)	40.23 (±2.48)	41.20 (±3.05)	**0.003**
	K2 (Steep K)	43.99 (±2.67)	43.53 (±2.22)	0.104
	Mean K	42.11 (±2.43)	42.36 (±2.49)	0.255
6	K1 (Flat K)	40.56 (± 2.30)	41.59 (±3.06)	**<0.001**
	K2 (Steep K)	44.39 (±2.57)	43.88 (±2.05)	0.111
	Mean K	42.47 (± 2.31)	42.74 (±2.45)	0.221
7	K1 (Flat K)	40.89 (±2.23)	41.83 (±3.13)	**<0.001**
	K2 (Steep K)	44.60 (±2.59)	43.97 (±1.91)	**0.037**
	Mean K	42.75 (±2.27)	42.90 (±2.47)	0.585

Supplementary Table S3: Detailed pre- and post-op corneal optical densitometry analysis

Zone (mm)	Depth	Pre-op Mean (±SD) (µm)	Post-op Mean (±SD) (µm)	P-Value
0-2	Anterior	28.55 (±16.08)	21.27 (±5.93)	0.056
	Centre	14.07 (±3.81)	12.65 (±2.54)	0.512
	Posterior	9.87 (±2.38)	9.37 (±1.48)	0.560
	Total	17.54 (±6.95)	14.43 (±3.05)	0.290
2-6	Anterior	29.57 (±12.30)	23.65 (±7.17)	0.064
	Centre	15.21 (±5.41)	13.49 (±4.23)	0.217
	Posterior	11.03 (±2.92)	10.06 (±2.45)	0.270
	Total	18.76 (±6.46)	15.73 (±4.27)	**<0.001**
6-10	Anterior	47.09 (±17.4)	39.85 (±14.86)	0.061
	Centre	26.74 (±10.25)	22.87 (±7.36)	0.538
	Posterior	18.53 (±5.77)	16.04 (±3.33)	0.341
	Total	30.79 (±10.90)	26.25 (±8.17)	**0.026**
10-12	Anterior	51.23 (±18.04)	42.44 (±11.99)	0.129
	Centre	28.64 (±7.46)	24.49 (±4.74)	0.191
	Posterior	20.54 (±4.71)	18.44 (±2.78)	0.348

	**Total**	33.47 (±9.66)	28.46 (±5.83)	**0.002**
**Overall cornea**	**Anterior**	37.57 (±13.41)	31.45 (±8.72)	0.053
	**Centre**	20.9 (±6.27)	18.13 (±4.59)	0.067
	**Posterior**	15.31 (±4.11)	13.22 (±2.10)	0.214
	**Total**	24.85 (±7.23)	20.94 (±4.9)	**<0.001**
